# Teachers, School Desks, and Lateral Curvature

**Published:** 1912-06-22

**Authors:** 


### THE IDEAL SCHOOL CLINIC.

### Teachers, School Desks, and Lateral Curvature.

One of the most common defects which the school doctor is called upon to deal with is scoliosis in weakly, lax musculatured children. The obvious prevailing causative factor in this condition is fatigue. It is usually fatigue ascribed to causes which are anything and everything except the real ones.

The school furniture, for example, is blamed, both by experts and by laymen, in a manner that makes the unprejudiced observer wonder how it is that since school desks and school benches are so absolutely horrible, all school children do not suffer from exaggerated forms of spinal curvature. As a matter of fact the direct influence of the desk and the bench has little effect in causing curvature. The main causative factor is fatigue which makes the child take up a bad position in order to throw the strain on to another set of muscles. In the majority of cases—excluding the small percentage in which there is a real osseous or ligamentous lesion present such as tuberculosis or osteomyelitis —the curves met with are fatigue curves, deformities due to tiredness.

Their prevention is, therefore, in the first place a matter for the teacher. The lessons and class work must be so arranged that there is an avoidance of sameness either of subject or of attitude. Cautious and experienced teachers will make their class stand up after each lesson, and will see that both boys and girls, and especially those in the infant classes, get proper relaxation after each subject before the next is tackled. Modern requirements of the syllabus of work necessitate certain modifications, but the teacher who takes a real and active interest in the welfare of his pupils will always find a way to arrange the day's work in such a manner that the maximum of accomplishment is allowed with the minimum of effort and consequently of fatigue.

### Teachers and Mental Strain.

Nor must it be forgotten that the fatigue engendering spinal curves may be both mental and physical or as much the former as the latter. This is a point very often overlooked by teachers who fail to realise the importance of the fact that mental and intellectual weariness inevitably promotes a laxity of the musculature. The physiological causes of this correlation between the two kinds of fatigue need not be detailed here, and, as a matter of fact, they are not perfectly known.

What is known is that the ergograph registers a noticeable difference between the mentally fatigued and the mentally energetic boy or girl just in the same way as "stimulus tests" show that there is a similar difference in response to stimuli between the physically tired and the ordinary pupil. In the infant classes these differences are much more striking and much more potent in their effects upon the child. The modern—and very excellent—fashion is to permit the small child to sleep and laze in the baby room, and the attitudes occupied by such babies are startling enough to one who maintains that posture, and not muscular weakness, produces scoliosis. If the postural theory were right nothing can be more harmful than to allow the babies to "lounge" on chair or desk or floor. But, again, as a matter of fact one finds, on careful examination, that the percentage of postural curves is much smaller in the infant than in any other department in schools where careful avoidance of fatigue is the rule. Undoubtedly the beginnings of real postural curves are laid in the lower departments in schools where no attention is paid to the precautions necessary to prevent fatigue.

### The Influence of School Furniture.

Probably the influence of school furniture in producing scoliosis is much over-estimated. In fact, school doctors who have paid close attention to the subject incline to think that in schools where the teachers see to it that fatigue is avoided and that the children take up natural attitudes during such work as writing, sewing, and drawing, the percentage of cases of scoliosis requiring treatment that are due to postural causes is very small. The teacher is largely responsible for the conditions where the percentage exceeds five. This may be taken as a good rule that works well in practice. If the school doctor finds a larger number of scholars suffering from lateral curvature he may take it for granted that the work done in the classes is of too strenuous and tiring a nature to be otherwise than detrimental to the health of the pupils. In that case he must take vigorous steps to bring about a reform.

Unfortunately in this country the power of the school doctor is very much limited. In Germany, for instance, he can order the whole class to attend special exercise drills, which take place at an adjoining clinic and which occupy the best part of the afternoon, so that the fatigue of the school work is largely avoided. Here with us he can only use his moral influence with the teachers. By pointing out, and proving to them, the harm done by fatigue he can often succeed in modifying the course of lessons. It is not always necessary to change the hours or the syllabus: all that is required is to arrange the subjects in such a fashion that undue strain is avoided and the lessons are made short and pleasant. For instance, in geography the children can be called out from their seats and the lesson given with all standing round globe or map, thus giving a change from the sitting attitude. Much personal attention is required on the part of the teacher, and in large classes such individual attention is almost impossible. For that reason small classes should be agitated for by school doctors, by teachers, and by parents.

